# Supplementary material for: Plasma Tie2 is a tumor vascular response biomarker for VEGF inhibitors in metastatic colorectal cancer
Source: Nat Commun. 2018 Nov 7;9:4672. doi: 10.1038/s41467-018-07174-1 (PMC6220185; doi:10.1038/s41467-018-07174-1)
Supplement: Supplementary file 1 — Supplementary Information [file 41467_2018_7174_MOESM1_ESM.pdf]

Supplementary information

Plasma Tie2 is a tumor vascular response biomarker for VEGF inhibitors in metastatic colorectal cancer

Jayson *et al* 2018

a.

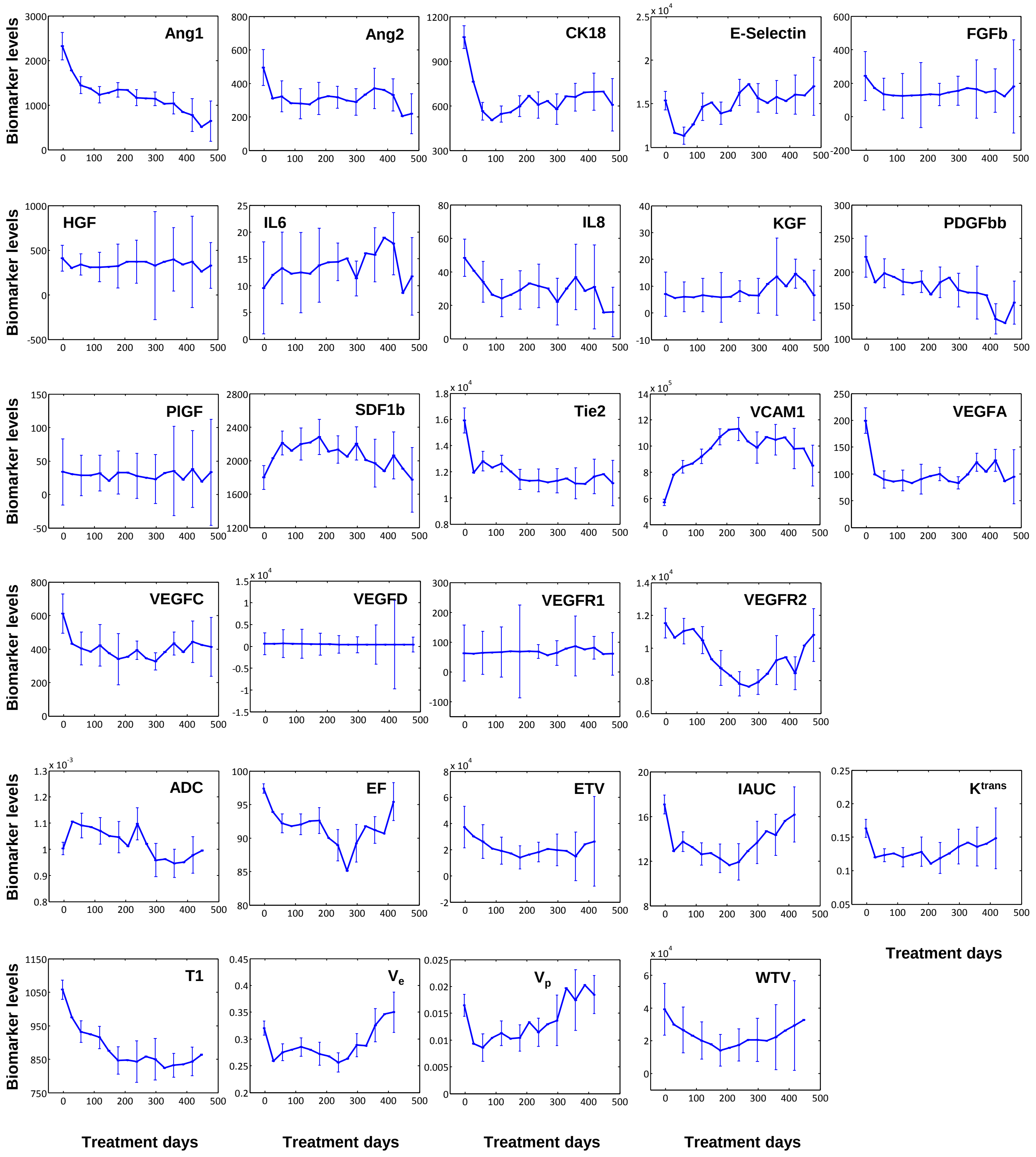

Supplementary Figure 1. Trajectories of circulating and imaging biomarkers

a. Unprocessed biomarker changes during treatment

The Y-axis log scale reduces the patient-to-patient variation observed in biomarker concentration. Error bars represent standard error (SE). The first 14 days were the period of single agent bevacizumab administration following which cytotoxic combination therapy was added. The number of patients at 0, 100, 200, 300, 400 and 500 days for circulating biomarkers was 70, 58, 42, 22 and 6 respectively and 70, 48, 44, 22 and 9 for imaging biomarkers.

This figure continues on the following two pages.

b.

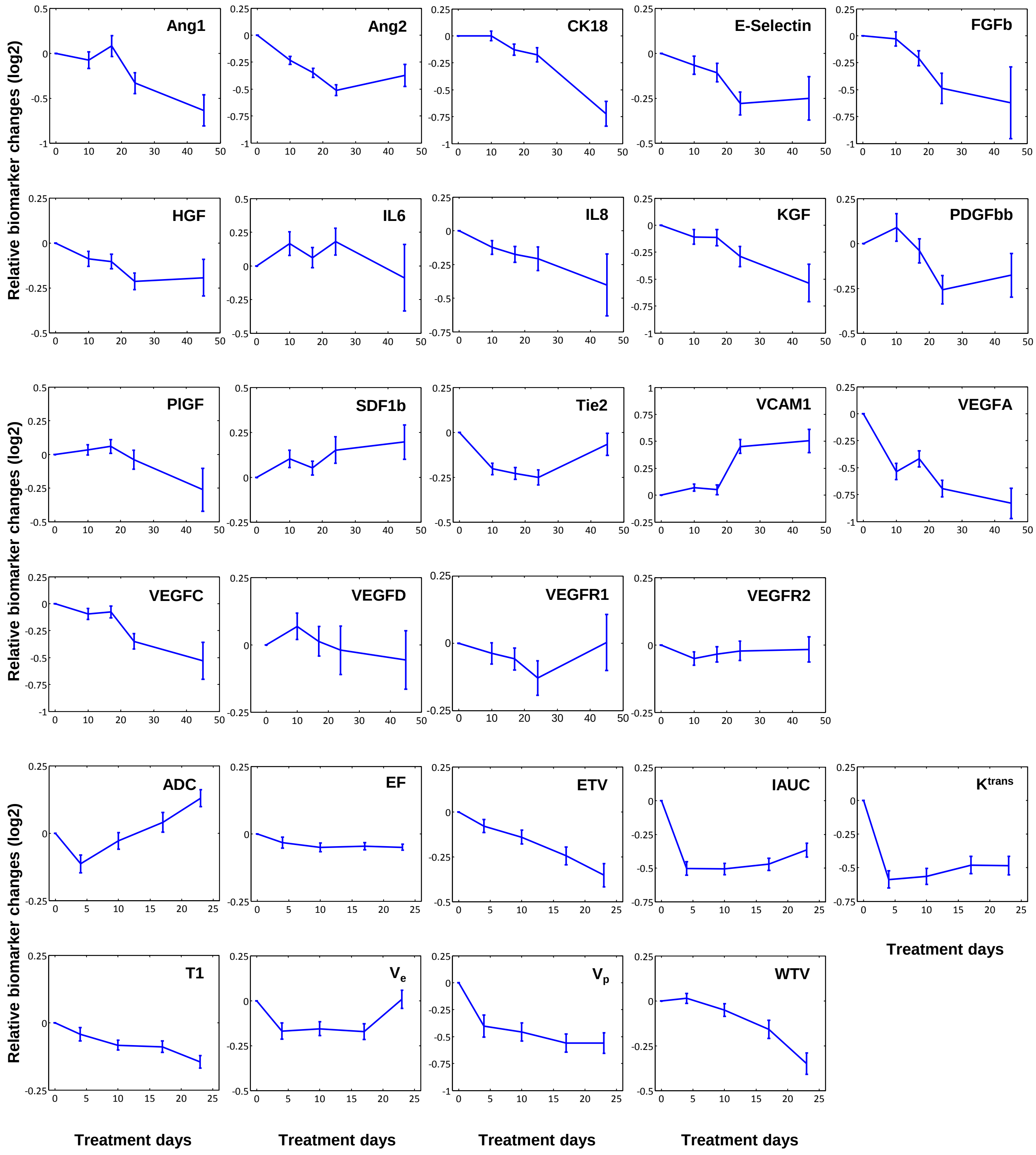

Supplementary Figure 1. Trajectories of circulating and imaging biomarkers

Continued from the previous page and continues on the following page.

b. Acute biomarker response to treatment (6 weeks after treatment started).

The Y-axis log scale reduces the patient-to-patient variation observed in biomarker concentration. Error bars represent standard error (SE).

The first 14 days were the period of single agent bevacizumab administration following which cytotoxic combination therapy was added.

c.

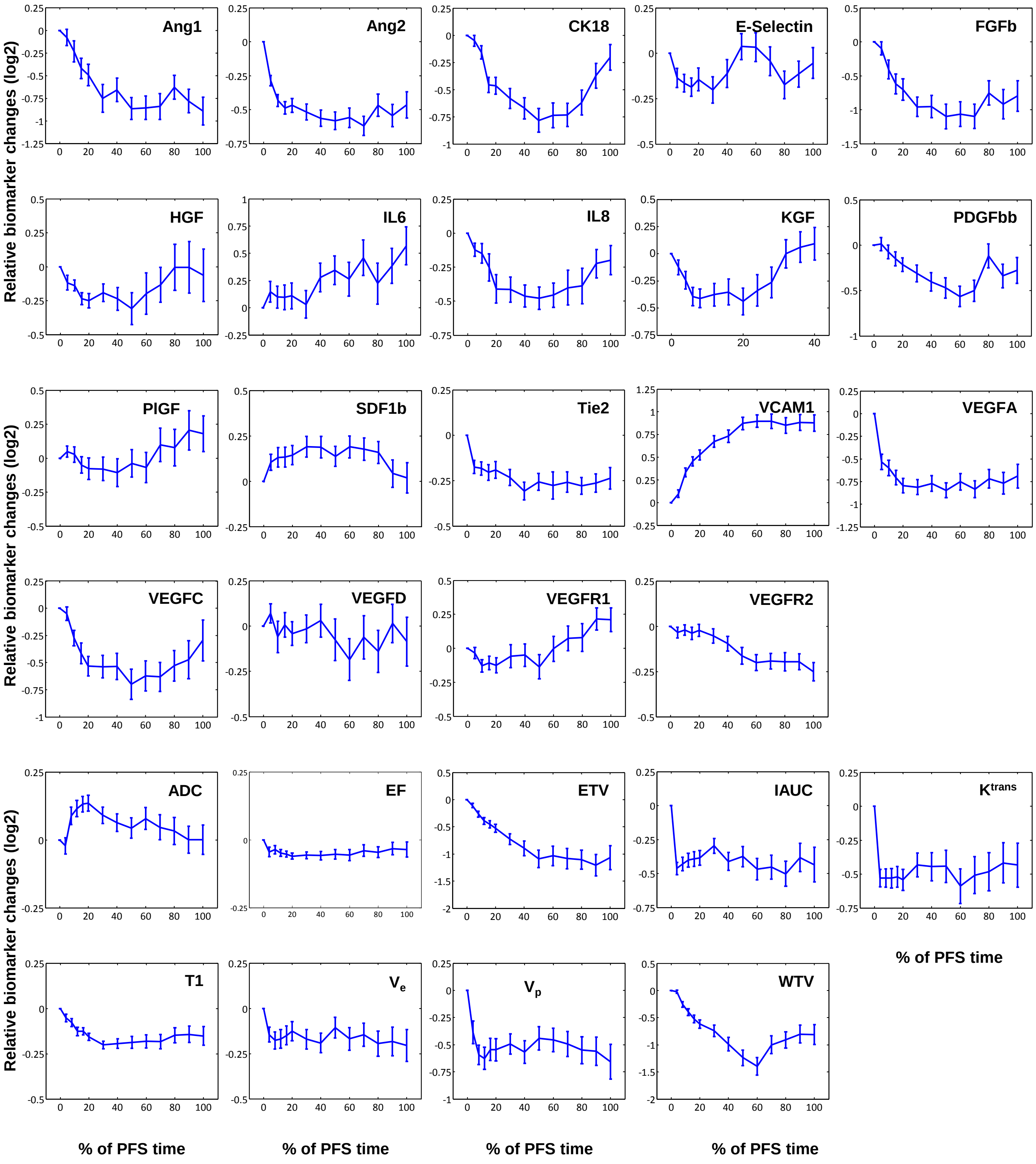

Supplementary Figure 1. Trajectories of circulating and imaging biomarkers

Continued from the previous two pages.

c. Change in biomarker from before treatment to progressive disease

The Y-axis log scale reduces the patient-to-patient variation observed in biomarker concentration and the X-axis the percentage of PFS interval allowing patients with different PFS intervals to be compared. (ranging from 0, where treatment started to 100, where the patient developed progressive disease). Error bars represent standard error (SE).

Ang1 and 2; angiopoietin 1 and 2, FGFb; fibroblast growth factor beta, HGF; hepatocyte growth factor, IL6 and 8; interleukins 6 and 8, KGF; keratinocyte growth factor, CK18; cytokeratin 18, PDGFbb; platelet-derived growth factor bb isoform, PlGF; placental growth factor, SDF1b; stromal-derived growth factor beta, VCAM1; vascular cell adhesion molecule 1; VEGFA, C, D, R1 and R2; vascular endothelial growth factor A, C and D and receptors 1 and 2, ADC; apparent diffusion coefficient, EF; ejection fraction, ETV; enhancing tumor volume, WTV; whole tumor volume, IAUC; initial area under the contrast agent concentration curve; Ktrans; endothelial transfer constant, Ve; extracellular extravascular space fractional volume, Vp; plasma fractional volume. PFS; progression-free survival.

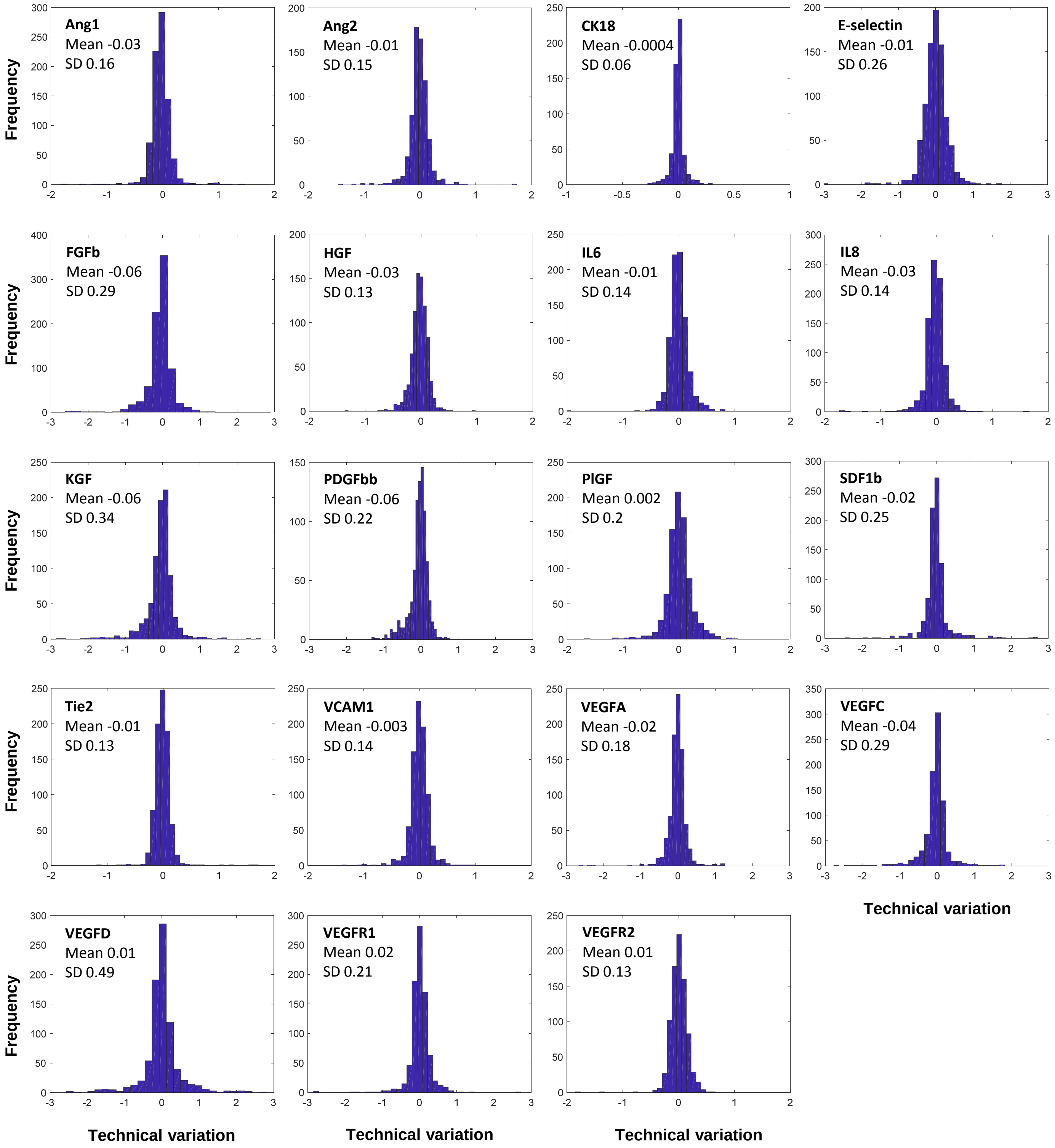

Supplementary Figure 2. Technical variation of ELISA used in the study

All circulating biomarkers were measured in duplicate using ELISAs; technical variation of the assays was estimated by log2 transformed ratio of the two technical replicate measurements. These histograms show the mean and standard deviations (SDs) of the technical variation. Ang1, Ang2, CK18, HGF, IL6, IL8, PlGF, Tie2, VEGFA, VCAM1 and VEGFR2 demonstrated the smallest variations in SDs of < 0.2. E-Selectin, FGFb, PDGFbb, SDF1b, VEGFC and VEGFR1 and demonstrated variations with SDs 0.2 - 0.3. KGF and VEGFD demonstrated variations with SDs >0.3.

Ang1 and 2; angiopoietin 1 and 2, FGFb; fibroblast growth factor beta, HGF; hepatocyte growth factor, IL6 and 8; interleukins 6 and 8, KGF; keratinocyte growth factor, CK18; cytokeratin 18, PDGFbb; platelet-derived growth factor bb isoform, PlGF; placental growth factor, SDF1b; stromal-derived growth factor beta, VCAM1; vascular cell adhesion molecule 1; VEGFA, C, D, R1 and R2; vascular endothelial growth factor A, C and D and receptors 1 and 2. ELISA; enzyme-linked immunosorbent assay.

a.

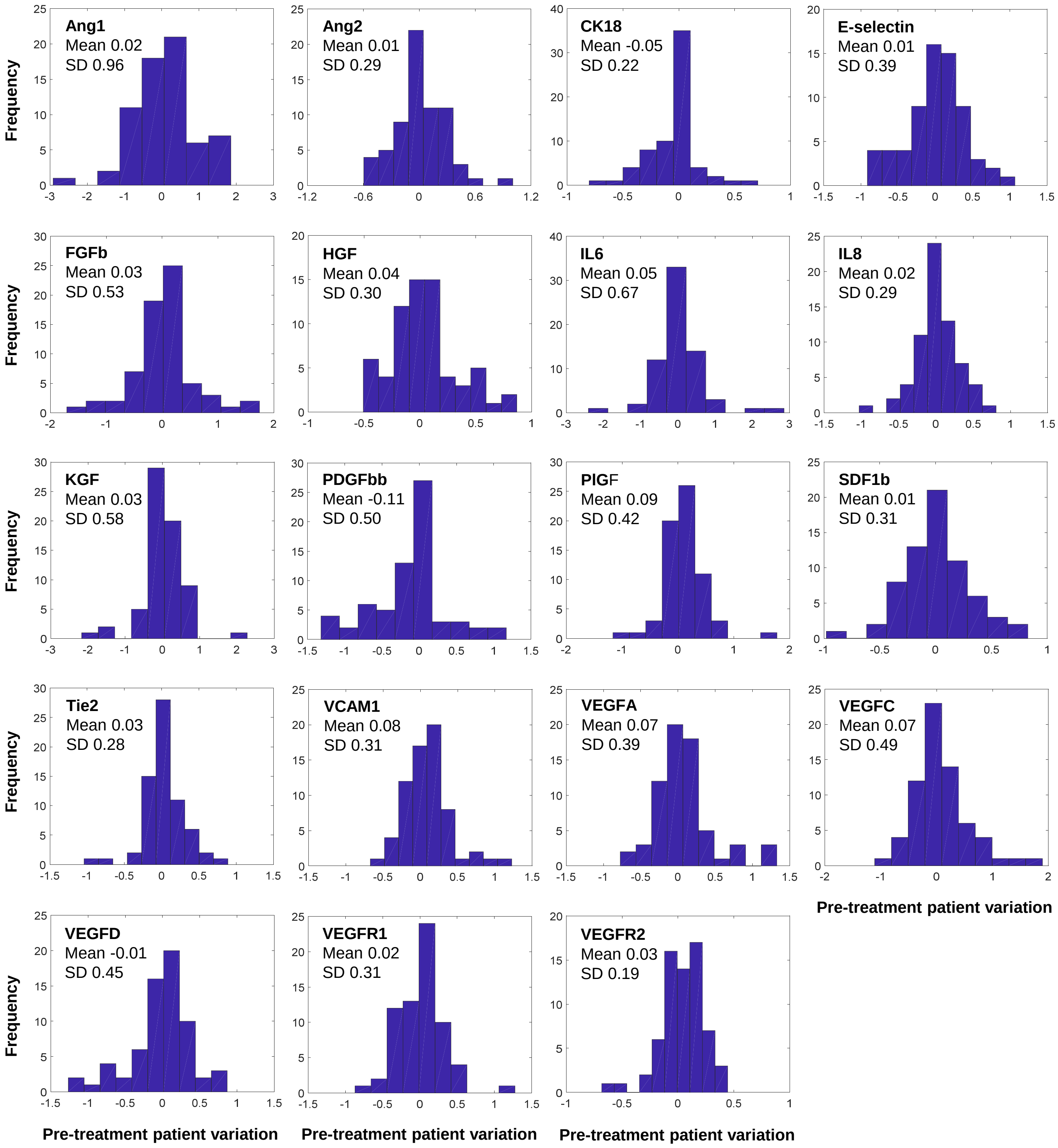

Supplementary Figure 3. Pre-treatment intra-patient variation of biomarkers

Intra-patient variation was examined for each biomarker using the two pre-treatment measurements.

a. Circulating biomarkers

Histograms of pre-treatment intra-patient variation were shown, with mean and standard deviation (SD) of technical variation using the log2 transformed ratio of the two measurements. Ang1, Ang2, CK18, IL8, HGF, Tie2 and VEGFR2 demonstrated the smallest variations with SDs < 0.3. E-Selectin, PDGFbb, PlGF, SDF1b, VCAM1, VEGFA, VEGFC, VEGFD and VEGFR1 demonstrated medium variations with SDs of 0.3 - 0.5. Ang1, FGFb, IL6 and KGF demonstrated largest variations with SD > 0.5. Fitted with Normal distributions, the figures indicated that the 95% CI of Tie2 and CK18 are $\pm$0.53 and $\pm$0.42 respectively.

This figure is continued on following page.

b.

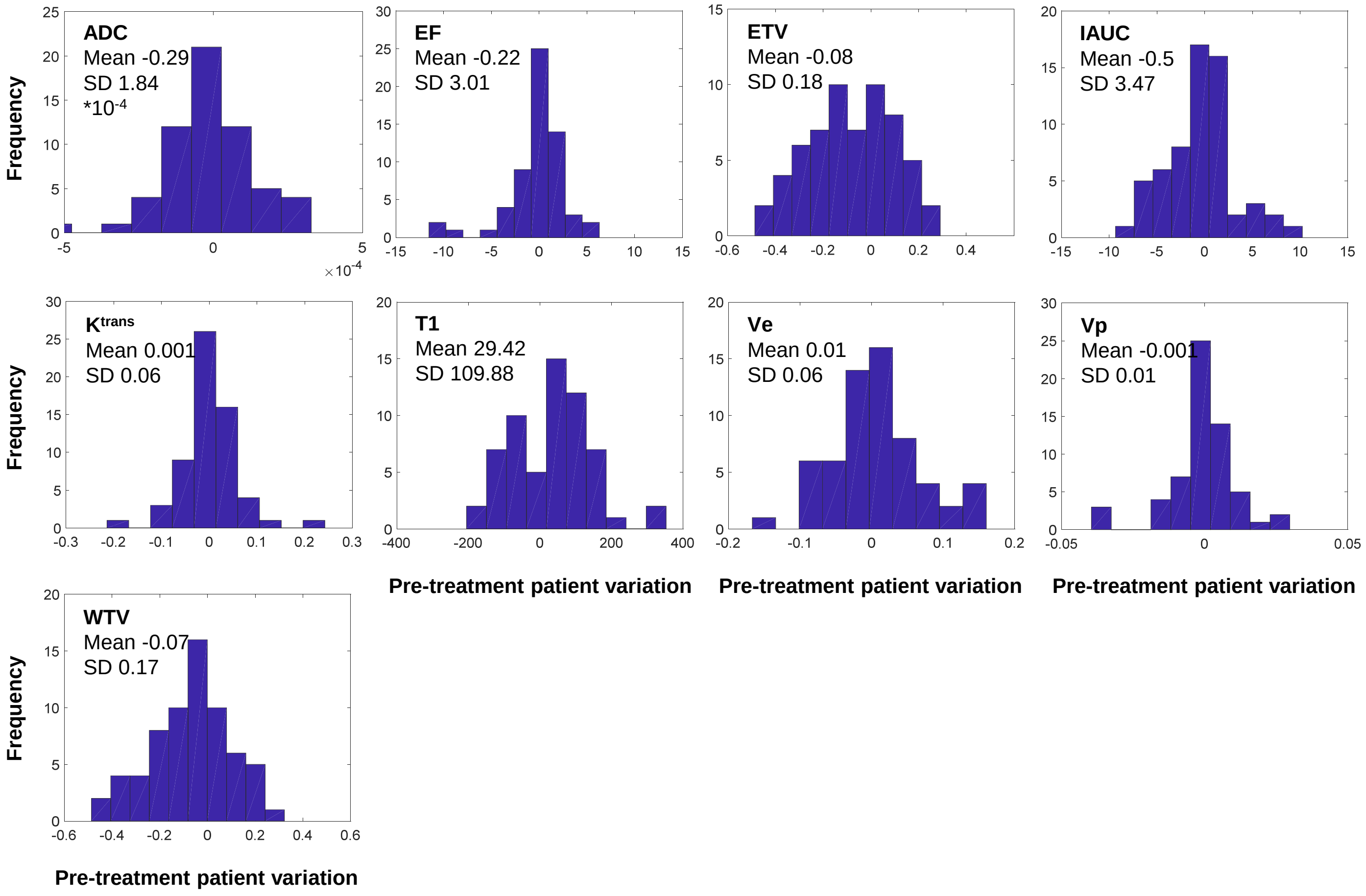

Supplementary Figure 3. Pre-treatment intra-patient variation of biomarkers

Continued from the previous page.

b. Imaging biomarkers

The variations of imaging WTV and ETV biomarkers were estimated for the log2 transformed ratio of the two measurements and other imaging biomarkers were estimated by their differences.

The pre-treatment variation in imaging biomarkers were difficult to compare with each other because they are measured in different units. However, WTV and ETV demonstrated superior repeatability with standard deviations smaller than any of the circulating biomarkers.

Ang1 and 2; angiopoietin 1 and 2, FGFb; fibroblast growth factor beta, HGF; hepatocyte growth factor, IL6 and 8; interleukins 6 and 8, KGF; keratinocyte growth factor, CK18; cytokeratin 18, PDGFbb; platelet-derived growth factor bb isoform, PlGF; placental growth factor, SDF1b; stromal-derived growth factor beta, VCAM1; vascular cell adhesion molecule 1; VEGFA, C, D, R1 and R2; vascular endothelial growth factor A, C and D and receptors 1 and 2, ADC; apparent diffusion coefficient, EF; ejection fraction, ETV; enhancing tumor volume, WTV; whole tumor volume, IAUC; initial area under the contrast agent concentration curve; Ktrans; endothelial transfer constant, Ve; extracellular extravascular space fractional volume, Vp; plasma fractional volume.

After 3 weeks of bevacizumab and one week of cytotoxic therapy

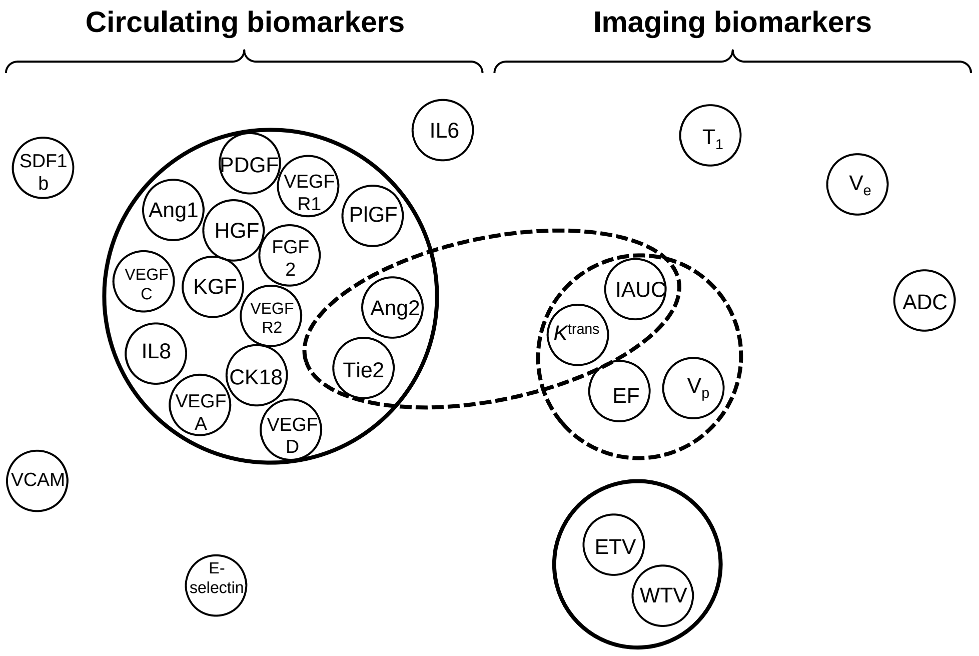

Supplementary Figure 4. Networks of circulating and imaging biomarkers

Pearson's correlation networks were constructed for circulating (left) and imaging biomarkers (right). The plots show correlation networks for biomarkers after 3 weeks of bevacizumab and a week of combination cytotoxic therapy. Clusters with median correlation coefficients above 0.5 are shown in thick black lines, while <0.5 but ≥ 0.35 are shown with dotted lines and correlations < 0.35 are not displayed.

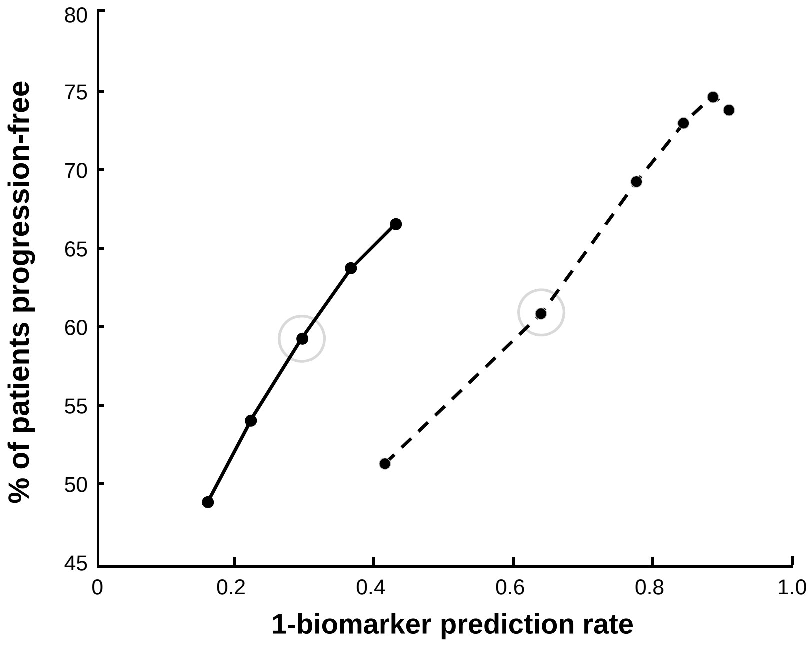

Supplementary Figure 5. Biomarker rules for predicting progression

The figure demonstrates the performance of using CK18 (dotted line) and Tie2 (solid line) as biomarkers for predicting tumor progression.

1 – biomarker prediction rate is plotted against percentage of PFS time when a prediction is made. Data points close to the top left corner indicate superior performance. The highlighted grey circles correspond to prediction rules as follows: if biomarkers are measured sequentially during treatment, 50% and 40% elevation of CK18 and Tie2 from their recorded nadir points indicate progression of the respective cancer tissue compartment.

CK18; cytokeratin 18.

	Sustained reduction of Tie2	Transient reduction of Tie2
Low VEGF-R2/ K^{trans}	23	7
High VEGF-R2/ K^{trans}	21	19

Supplementary Table 1. Association between VEGF-R2/K^{trans} and Tie2

The table lists the number of patients that belonged to categories defined by pre-treatment [VEGF-R2:K^{trans}] and Tie2 trajectories during treatment. Categorization of Tie2 trajectories was carried out using an unsupervised hierarchical clustering approach (Figure 3) and the proportion of resulting clusters was used to determine the cut-off for VEGF-R2/K^{trans} categorization. A chi-squared test was carried out on the above contingency table, resulting in a p-value = 0.014 indicating that patients with high VEGF-R2/K^{trans} ratios, pre-treatment, were more likely to manifest a transient reduction of Tie2.

		Day							
	Pre-treatment	1	3	8	15	22	6 weeks	6 months	PD
Anatomical CT[1]	✓								✓
Bevacizumab[2]		✓			✓				
FOLFOX 6[2]					✓				
Circulating biomarkers[3]	✓✓		✓	✓	✓	✓	✓	✓	✓
Advanced MRI[4]	✓✓		✓	✓	✓	✓		✓	✓

Supplementary Table 2. Schedule of biomarkers and treatments

[1] Anatomical CT scanning was performed every 3 months, [2]Following single agent bevacizumab for the first 2 weeks, patients received bevacizumab with FOLFOX6 or CAPOX until progression, [3]Circulating biomarkers were taken twice before treatment to establish reproducibility, as shown in the table and then every 6 weeks until progression, [4] DCE-MRI and DWI were performed as shown above.

PD; progressive disease, FOLFOX6; leucovorin calcium (folinic acid), fluorouracil, and oxaliplatin, CAPOX; capecitabine and oxaliplatin, DCE-MRI; dynamic contrast-enhanced magnetic resonance imaging, DWI; diffusion-weighted imaging.
